# Supplementary material for: H2A.Z deposition by SWR1C involves multiple ATP-dependent steps
Source: Nat Commun. 2022 Nov 17;13:7052. doi: 10.1038/s41467-022-34861-x (PMC9672302; doi:10.1038/s41467-022-34861-x)
Supplement: Supplementary file 1 — Supplementary Information [file 41467_2022_34861_MOESM1_ESM.pdf]

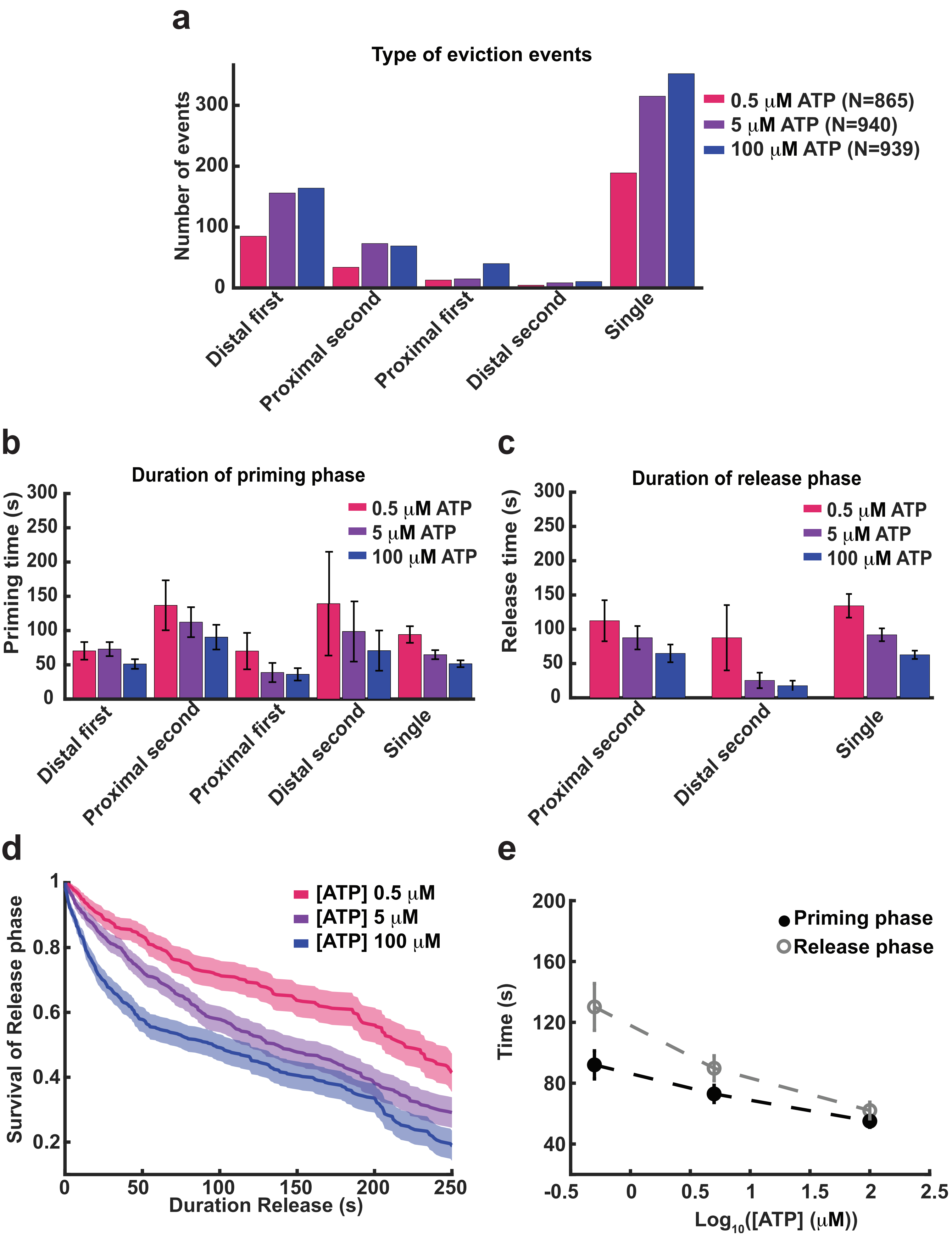
a
Type of eviction events
Number of events
0.5 μM ATP (N=865)
5 μM ATP (N=940)
100 μM ATP (N=939)
Distal first
Proximal second
Proximal first
Distal second
Single
b
Duration of priming phase
Priming time (s)
0.5 μM ATP
5 μM ATP
100 μM ATP
c
Duration of release phase
Release time (s)
d
Survival of Release phase
Duration Release (s)
[ATP] 0.5 μM
[ATP] 5 μM
[ATP] 100 μM
e
Time (s)
Priming phase
Release phase
Log10([ATP] (μM))

Supplementary Fig. 1. The number of eviction events and duration of the priming and release phase is dependent on ATP concentration. (**a**) Observed events for each type of eviction with indicated ATP concentration, 100 μM ATP N=635 observed events for 939 nucleosomes from 8 replicates, 5 μM ATP N=569 observed events for 940 nucleosomes from 8 replicates, 0.5 μM ATP N=325 observed events for 865 nucleosomes from 7 replicates. (**b**) Duration of priming phase for each type of eviction obtained from exponential fit of the distribution, error bars represent C.I. = 95 % confidence interval of exponential fit for the indicated ATP concentration, 100 μM ATP Distal first N=164, Proximal second N=69, Proximal first N=40, Distal second N=10, and Single N=352 observed events for 939 nucleosomes, 5 μM ATP Distal first N=156, Proximal second N=73, Proximal first N=15, Distal second N=10, and Single N=315 observed events for 940 nucleosomes, 0.5 μM ATP Distal first N=85, Proximal second N=34, Proximal first N=13, Distal second N=4, and Single N=189 observed events for 865 nucleosomes. (**c**) Duration of release phase for each type of eviction correspond to the half-life obtained from the Kaplan-Meier estimate and error bars represent 95 % C.I. for the indicated ATP concentration, 100 μM ATP Proximal second N=69, Distal second N=10, and Single N=352 observed events for 939 nucleosomes, 5 μM ATP Proximal second N=73, Distal second N=10, and Single N=315 observed events for 940 nucleosomes, 0.5 μM ATP, Proximal second N=34, Distal second N=4, and Single N=189 observed events for 865 nucleosomes (**d**) Release phase survival kinetics (Kaplan-Meier estimate) for 100 μM ATP (N=431 observed for 939 nucleosomes from 8 replicates) (blue), 5 μM ATP (N=398 observed for 940 nucleosomes from 8 replicates) (purple) 0.5 μM ATP (N=227 observed for 865 nucleosomes from 7 replicates) (magenta). The x axis indicates survival time for the fraction of molecules yet to dissociate from SWR1C-nucleosome complex. Solid line represents the fit from Kaplan-Meier estimate. Shaded areas, 95%

confidence intervals. **(e)** Duration of the priming and release phase aggregating all events. The x axis indicates ATP concentration (**b,e**) Priming duration values derived from fitting distributions to a single exponential and error bars represent 95 % C.I, (**c,d,e**) the release duration values where obtained from the Kaplan-Meier estimate and error bars represent 95 % C.I.

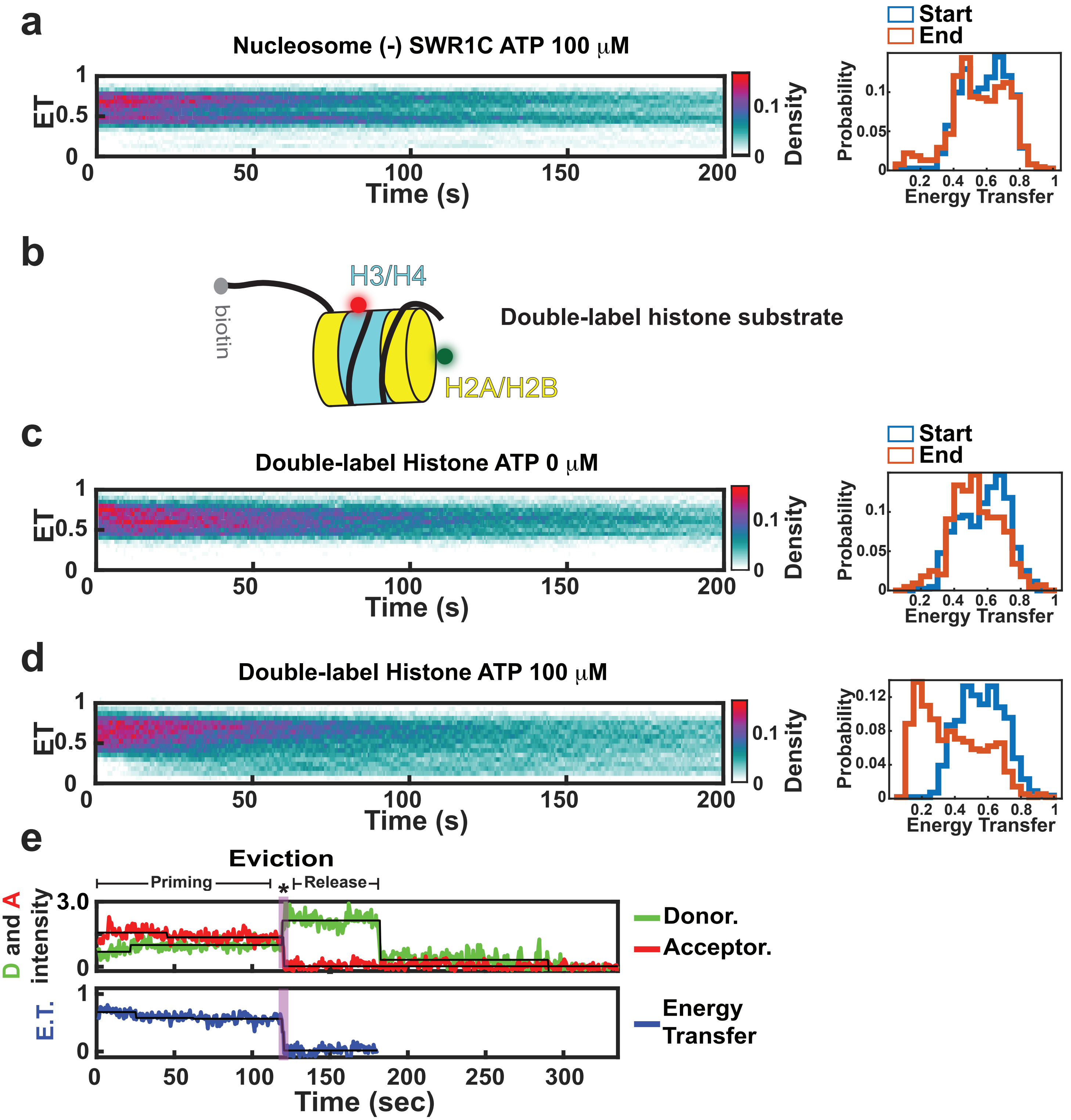
a
Nucleosome (-) SWR1C ATP 100 μM
ET
Time (s)
Density
Start
End
Probability
Energy Transfer
b
H3/H4
biotin
Double-label histone substrate
H2A/H2B
c
Double-label Histone ATP 0 μM
ET
Time (s)
Density
Start
End
Probability
Energy Transfer
d
Double-label Histone ATP 100 μM
ET
Time (s)
Density
Probability
Energy Transfer
e
Eviction
Priming
Release
D and A intensity
Donor.
Acceptor.
E.T.
Energy Transfer
Time (sec)

Supplementary Fig. 2. Additional controls for smFRET analysis of SWR1C-mediated H2A eviction. (**a**) Kymograph (left) and histogram (right) analysis showing stability of the nucleosome substrate in absence of SWR1C and ATP. This substrate harbors fluorophores on the linker distal DNA end and H2A. (**b**). Schematic of double-label histone substrate where the FRET pair is located on histones H3 and H2A. (**c,d**). Kymographs (left) and histograms (right) for reactions containing the double-label histone substrate, SWR1C, and either no ATP (**c**) or 100 micromolar ATP (**d**). **(e).** Representative smFRET trajectory for a reaction containing the double-label histone substrate, SWR1C, and 100 micromolar ATP. Note rapid loss of FRET indicating eviction (*) from nucleosome follows a priming phase. Post eviction there is a low FRET release phase before loss of donor signal that indicates dissociation of H2A-H2B from the nucleosome.

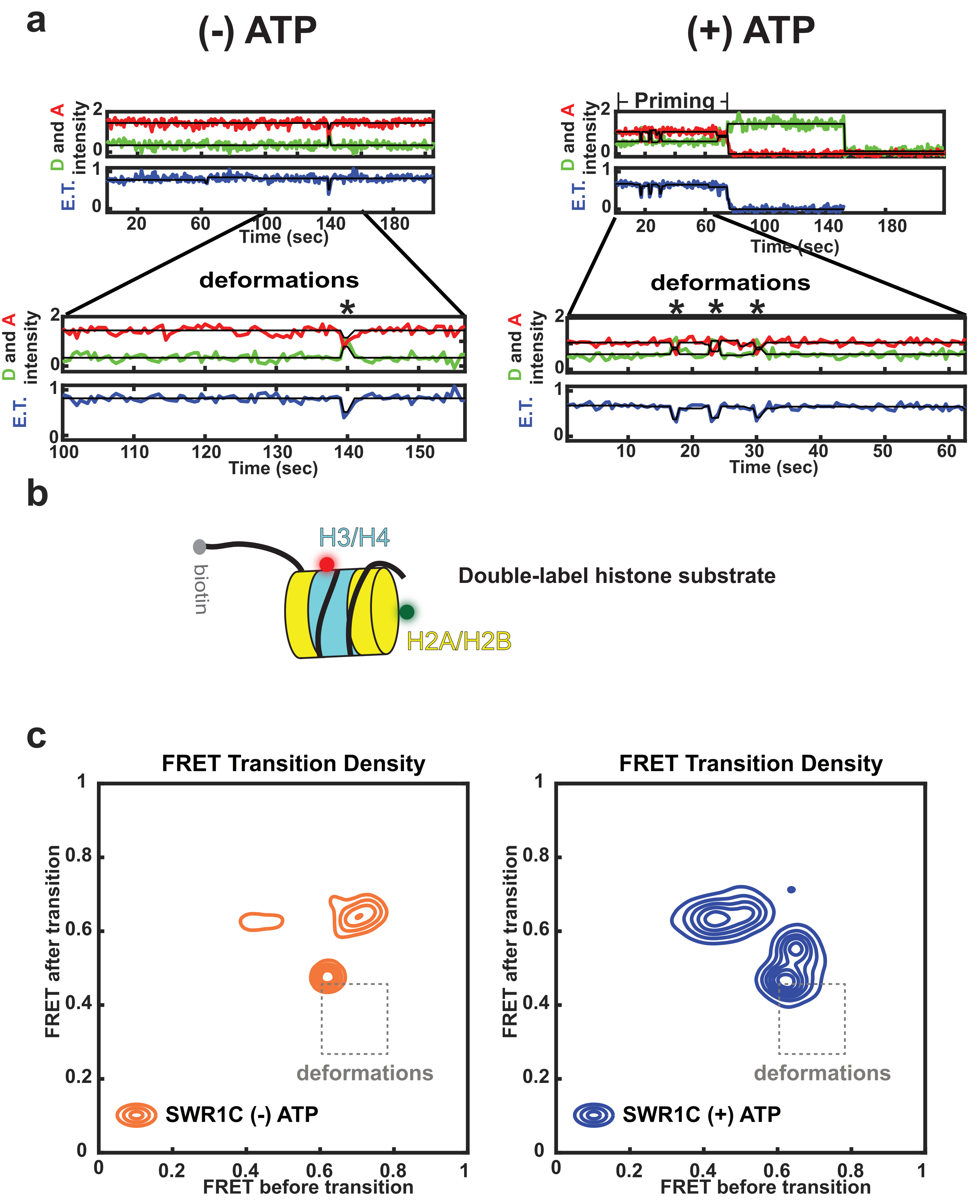
a
(-) ATP
(+) ATP
Priming
D and A intensity
E.T.
Time (sec)
deformations
*
b
H3/H4
biotin
H2A/H2B
Double-label histone substrate
c
FRET Transition Density
FRET after transition
FRET before transition
deformations
SWR1C (-) ATP
SWR1C (+) ATP

Supplementary Fig. 3. The priming phase for a substrate with H3 and H2A FRET probes shows transient FRET fluctuations. (**a**). Sample trajectories of the priming phase for reactions containing SWR1C in the absence or presence of ATP. * denote transient FRET fluctuations that likely correspond to octamer deformations. (**b**). Schematic of nucleosomal substrate. (**c**). Priming phase E.T. transition density plots. Double-label histone substrate with SWR1C. The intensity of E.T. transitions are normalized to the total observation window for 0 μM ATP (N=65) (left) and 100 μM ATP (N=78) (right).

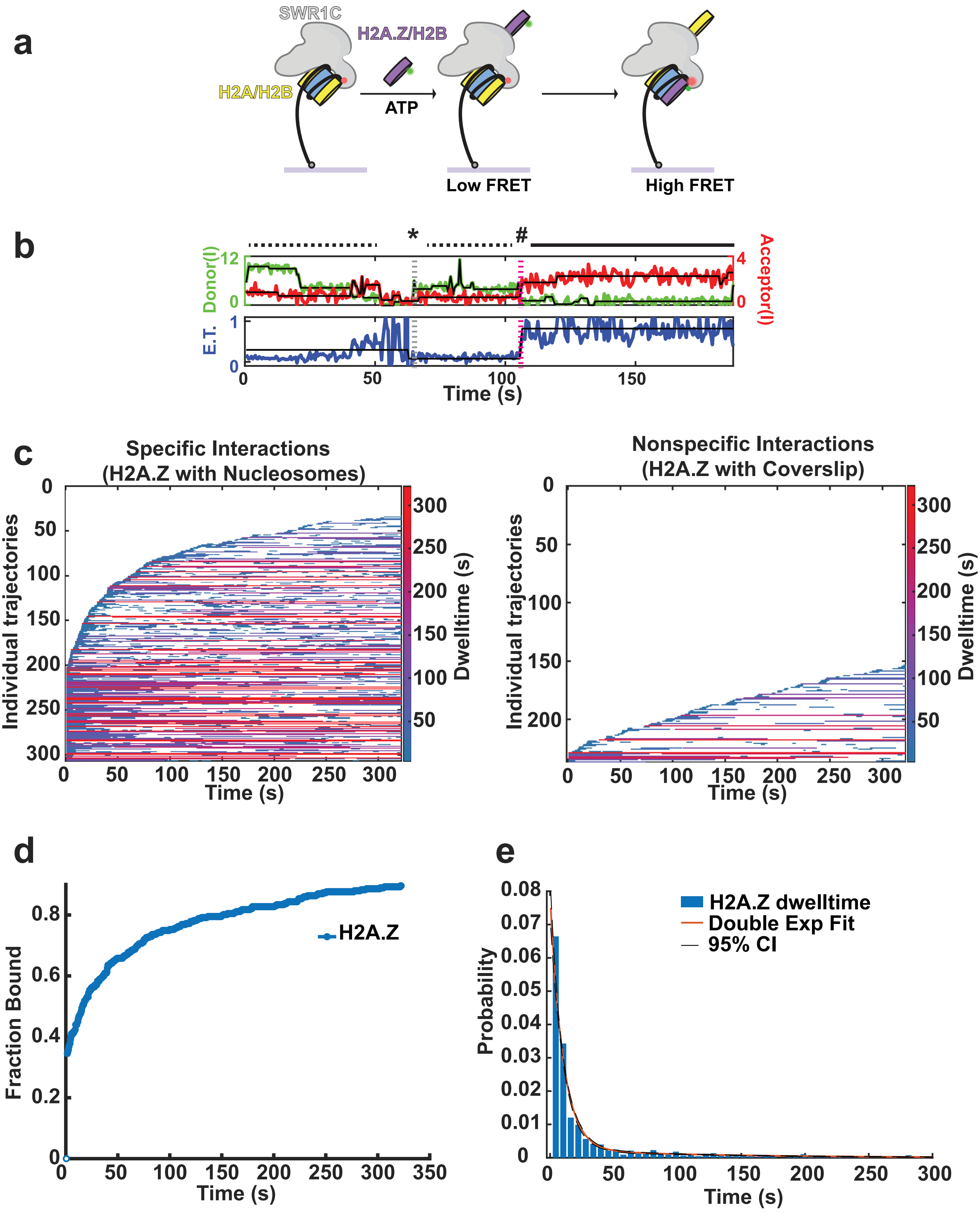
a
SWR1C
H2A.Z/H2B
H2A/H2B
ATP
Low FRET
High FRET
b
*
#
Donor(I)
Acceptor(I)
E.T.
Time (s)
c
Specific Interactions
(H2A.Z with Nucleosomes)
Nonspecific Interactions
(H2A.Z with Coverslip)
Individual trajectories
Dwelltime (s)
Time (s)
d
H2A.Z
Fraction Bound
Time (s)
e
H2A.Z dwelltime
Double Exp Fit
95% CI
Probability
Time (s)

Supplementary Fig. 4. SWR1C binds and deposits H2A.Z within the nucleosome (**a**) Schematic of the smFRET H2A.Z deposition assay. (**b**) Example trajectory highlighting binding events of H2A.Z (dotted line), association event preceding deposition (*) and deposition (solid line) (#). The H2A.Z labeled with Cy3B donor fluorophore was excited, and donor emission (green) and nucleosome labeled with ATTO 647N acceptor emission (red) were recorded (top panel) and used to calculate energy transfer efficiency (blue, bottom panel). (**c**) (right side) Rastergrams summarize colocalization traces of individual SWR1C-nucleosome complexes (N=307) each in a single row and sorted according to the arrival time of H2A.Z. (left side) Rastergrams summarize traces for individual background ROI (N=287). The events are colored by dwell time. (**d**) Fraction of SWR1C-nucleosome complexes that are bound by H2A.Z sorted by time arrival time. (**e**) Distribution of H2A.Z dwelltime, red line represents the fit to double exponential, and black dashed line corresponds to 95 % C.I. from bootstrap analysis.

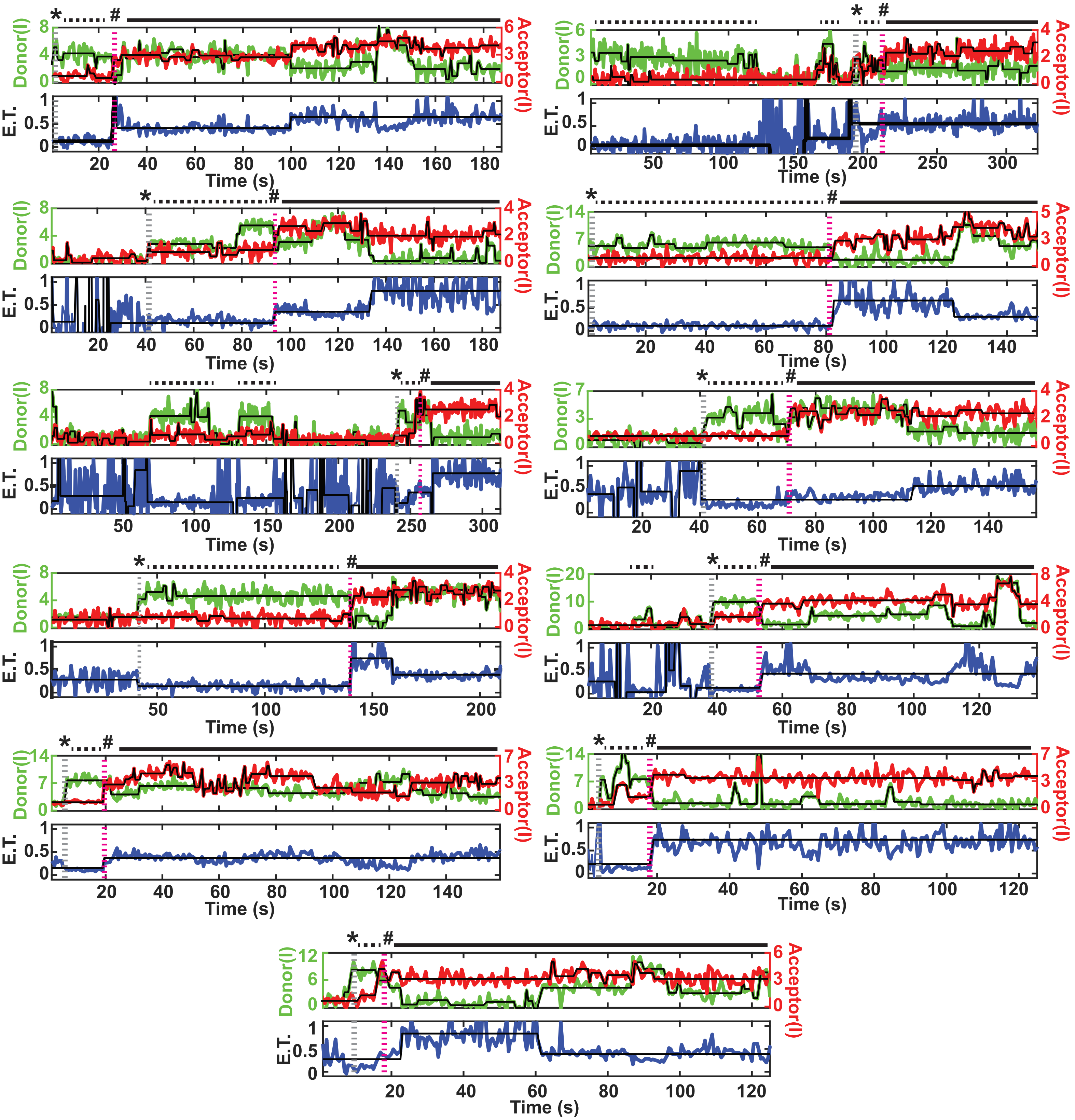
Donor(I)
Acceptor(I)
E.T.
Time (s)

Supplementary Fig. 5. Sample single-molecule colocalization and deposition of H2A.Z with the SWR1C-nucleosome complex, related to Supplementary Fig. 4. Example trajectory highlighting binding events of H2A.Z (dotted line), association event preceding deposition (*) and deposition (solid line) (#). The H2A.Z labeled Cy3B donor fluorophore was excited, and donor emission (green) and nucleosome labeled ATTO 647N acceptor emission (red) were recorded (top panel) and used to calculate energy transfer efficiency (blue, bottom panel).

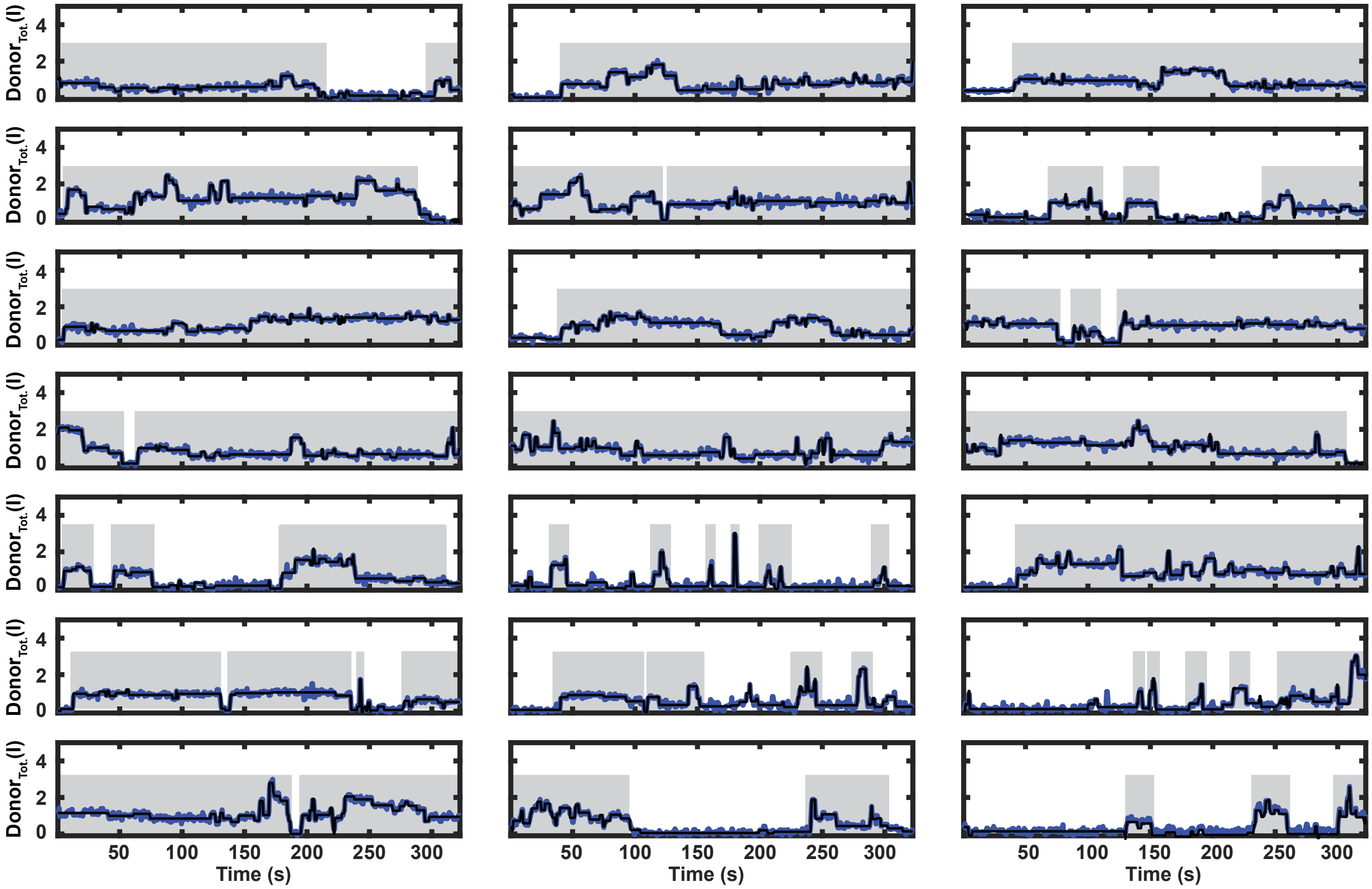

Donor$_{Tot.}$(I)
0
2
4
50
100
150
200
250
300
Time (s)

Supplementary Fig. 6. Sample single-molecule colocalization of H2A.Z with the SWR1C-nucleosome complex, related to Supplementary Fig. 4. Example trajectory highlighting binding events of H2A.Z (shaded areas). The H2A.Z labeled Cy3B donor fluorophore was excited, and total donor emission (blue) was calculated as the sum of donor emission and donor excited acceptor emission and normalized to mean intensity for H2A.Z colocalization.

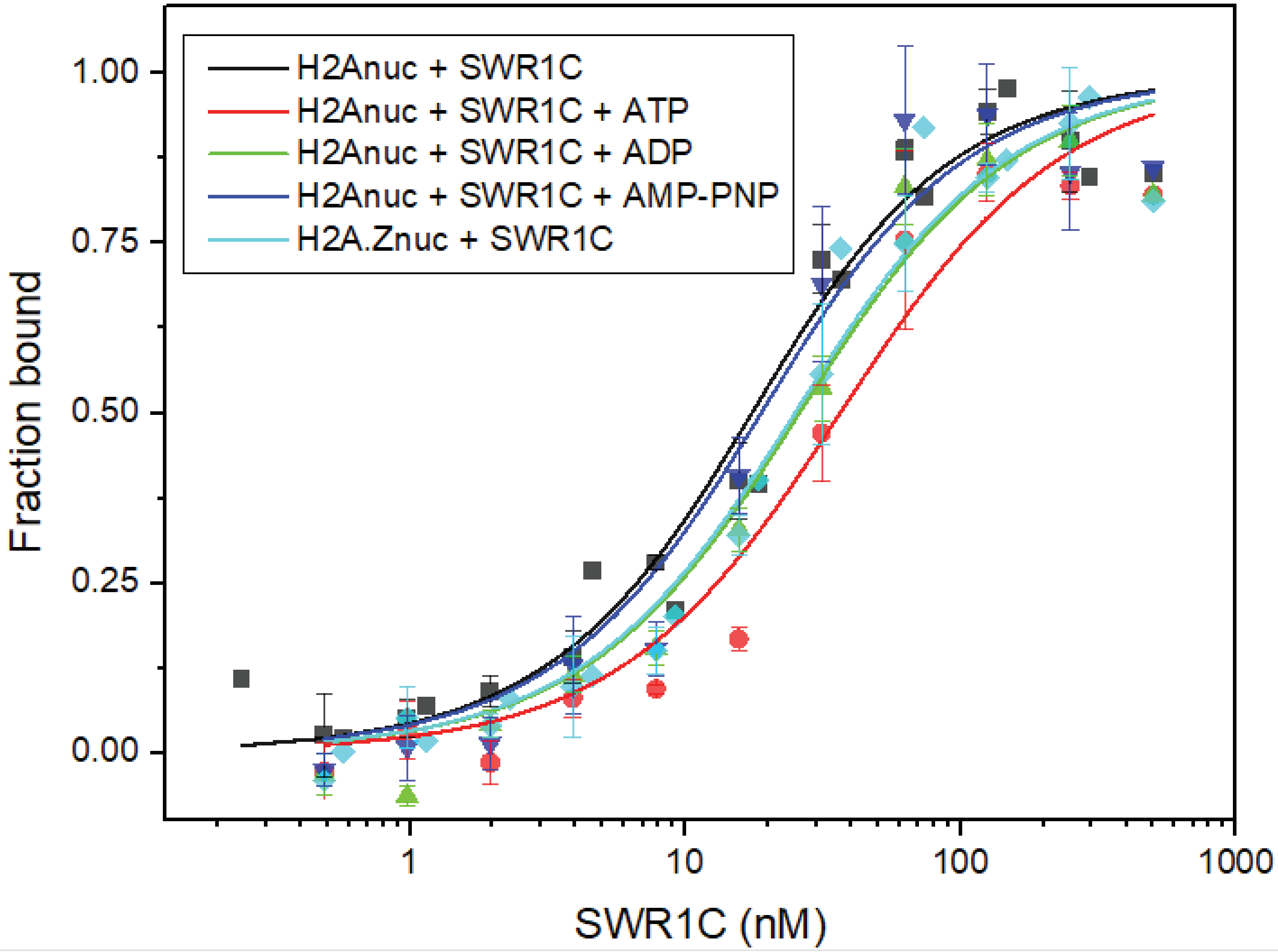

H2Anuc + SWR1C
H2Anuc + SWR1C + ATP
H2Anuc + SWR1C + ADP
H2Anuc + SWR1C + AMP-PNP
H2A.Znuc + SWR1C
Fraction bound
1.00
0.75
0.50
0.25
0.00
1
10
100
1000
SWR1C (nM)

Supplementary Fig. 7. Normalized fluorescence polarization plots and fitted curves for titrated SWR1C binding to an H2A nucleosome in the presence or absence of the indicated nucleotides. Each curve represents a global fit of 3 replicates with the standard error indicated by the error bars (+/- SEM). The concentration of SWR1C on the X-axis is displayed on a log_{10}-scale.

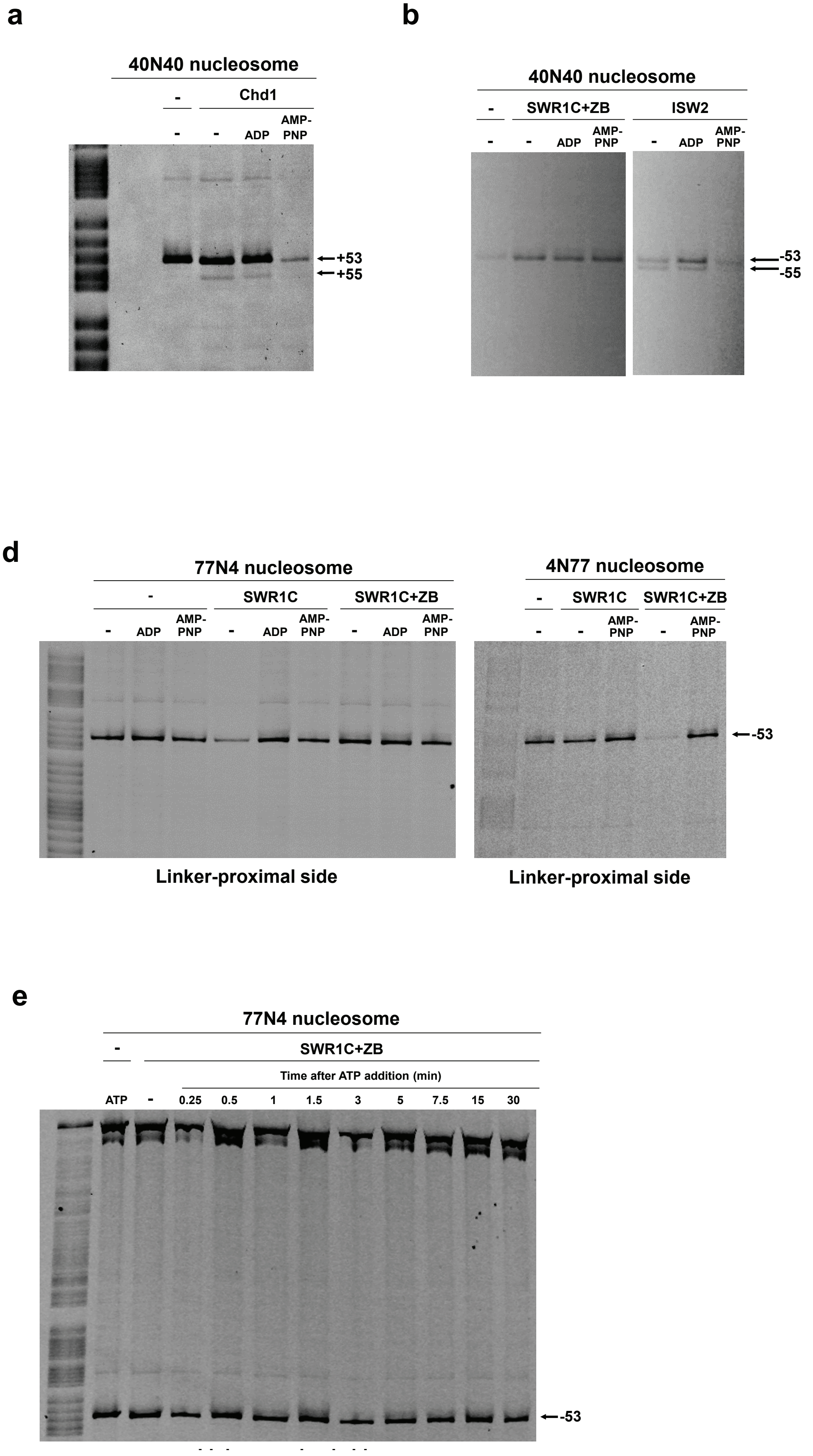
a
40N40 nucleosome
-
Chd1
-
-
ADP
AMP-PNP
+53
+55
b
40N40 nucleosome
-
SWR1C+ZB
ISW2
-
-
ADP
AMP-PNP
-
ADP
AMP-PNP
-53
-55
c
4N77 nucleosome
-
SWR1C
SWR1C+ZB
-
-
AMP-PNP
-
AMP-PNP
+53
Linker-distal side
d
77N4 nucleosome
-
SWR1C
SWR1C+ZB
-
ADP
AMP-PNP
-
ADP
AMP-PNP
-
ADP
AMP-PNP
Linker-proximal side
4N77 nucleosome
-
SWR1C
SWR1C+ZB
-
-
AMP-PNP
-
AMP-PNP
-53
Linker-proximal side
e
77N4 nucleosome
-
SWR1C+ZB
Time after ATP addition (min)
ATP
-
0.25
0.5
1
1.5
3
5
7.5
15
30
-53

Supplementary Fig. 8. Site-directed DNA-histone mapping on different nucleosome templates. (**a**) Chd1 binding in apo or ADP-bound state induces a 2-nt shift at SHL5.5 on a 40N40 nucleosome. (**b**) SWR1C does not translocate DNA on the opposite SHL5 of the 40N40 nucleosome relative to the side shown in Figure 6A. Isw2 binding induces a 2-nt shift at SHL5.5 in Apo and ADP-bound state. (**c**) SWR1C does not alter the DNA path near its predicted binding site on the linker-distal side of a 4N77 nucleosome. (**d**) SWR1C does not change the path of nucleosomal DNA on the linker-proximal side of an asymmetric nucleosome regardless of 601 positioning sequence asymmetry. (**e**) SWR1C does not change the path of nucleosomal DNA on the linker-proximal side during an ATP-dependent exchange reaction. Crosslinking reactions with SWR1C, Chd1, and Isw2 were repeated with at least two biological replicas, yielding similar results.

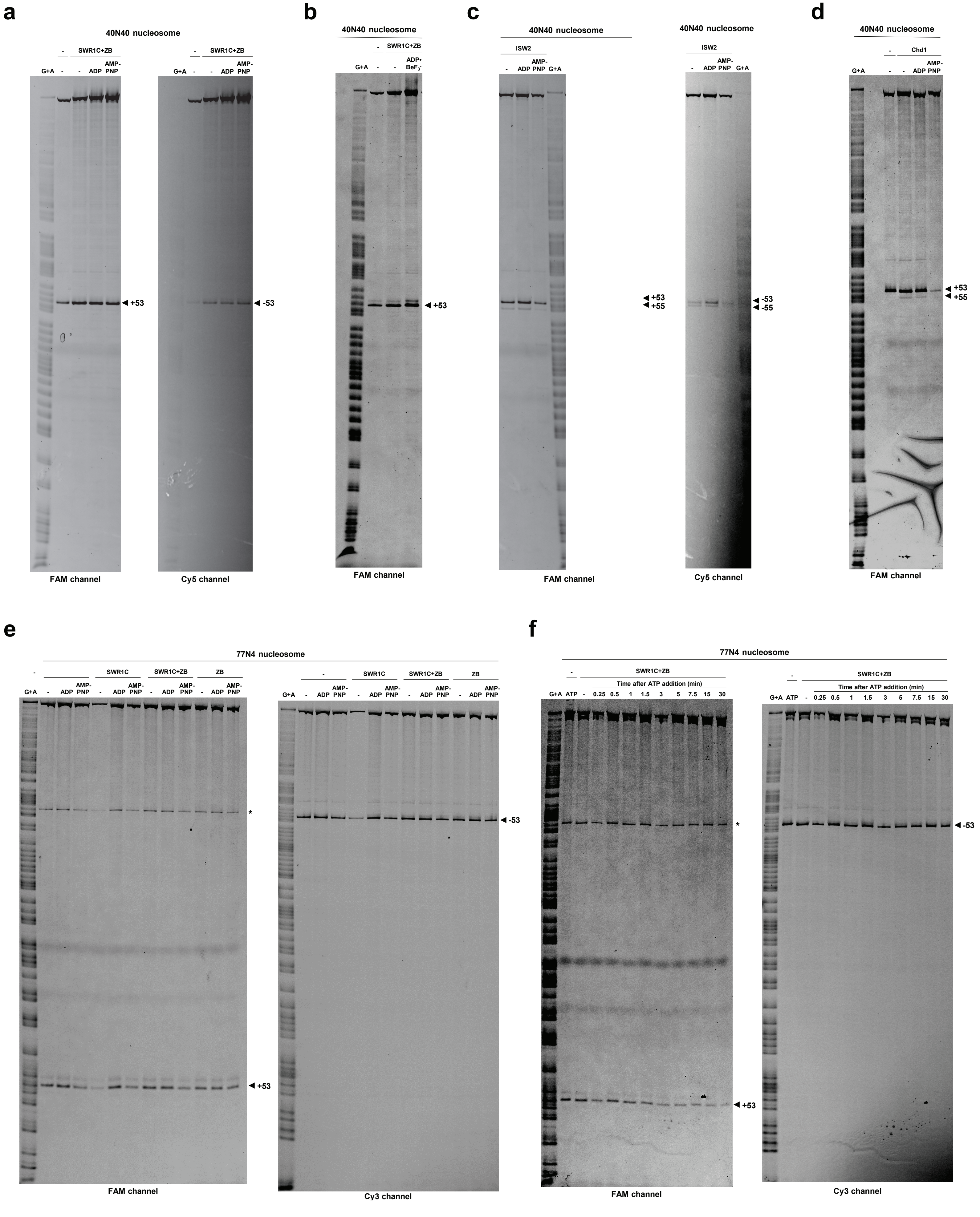
a
40N40 nucleosome
-
SWR1C+ZB
G+A
-
-
ADP
AMP-PNP
◀ +53
◀ -53
FAM channel
Cy5 channel
b
40N40 nucleosome
-
SWR1C+ZB
G+A
-
-
ADP•BeF3-
◀ +53
FAM channel
c
40N40 nucleosome
ISW2
-
ADP
AMP-PNP
G+A
◀ +53
◀ +55
◀ -53
◀ -55
FAM channel
Cy5 channel
d
40N40 nucleosome
-
Chd1
G+A
-
-
ADP
AMP-PNP
◀ +53
◀ +55
FAM channel
e
77N4 nucleosome
-
SWR1C
SWR1C+ZB
ZB
G+A
-
ADP
AMP-PNP
*
◀ +53
◀ -53
FAM channel
Cy3 channel
f
77N4 nucleosome
-
SWR1C+ZB
Time after ATP addition (min)
G+A
ATP
-
0.25
0.5
1
1.5
3
5
7.5
15
30
*
◀ +53
◀ -53
FAM channel
Cy3 channel

g

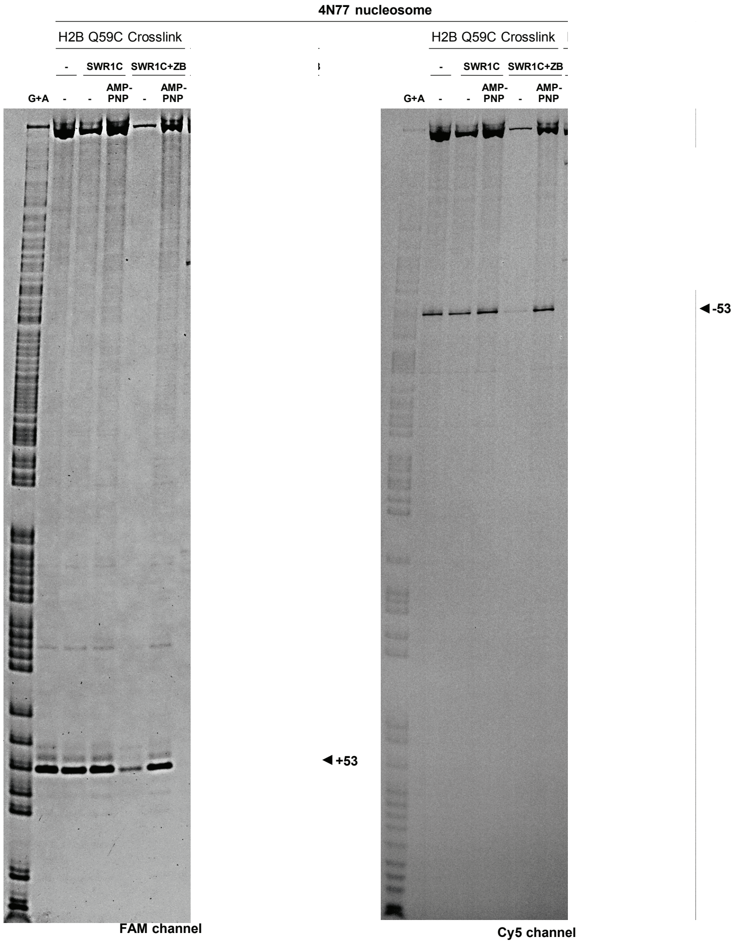
4N77 nucleosome
H2B Q59C Crosslink
-
SWR1C
SWR1C+ZB
G+A
-
-
AMP-PNP
-
AMP-PNP
◄+53
FAM channel
H2B Q59C Crosslink
-
SWR1C
SWR1C+ZB
G+A
-
-
AMP-PNP
-
AMP-PNP
◄-53
Cy5 channel

Supplementary Fig. 9. Full sequencing gel images for DNA-histone mapping in Fig. 6 and Supplementary Fig. 8. (**a**) Extended gel of Fig. 6b left panel (left) and Supplementary Fig. 8b left panel (right) scanning at 473 nm and 635 nm, respectively. (**b**) Extended gel of Fig. 6b middle panel scanning at 473 nm. (**c**) Extended gel of Fig. 6b right panel (left) and Supplementary Fig. 8b (right) scanning at 473 nm and 635 nm, respectively. (**d**) Extended gel of Supplementary Fig. 8a scanning at 473 nm. (**e**) Extended gel of Fig. 6c (left) and Supplementary Fig. 8d left panel (right) scanning at 473 nm and 532 nm, respectively. (**F**) Extended gel of Fig. 6d (left) and Supplementary Fig. 8e (right) scanning at 473 nm and 635 nm, respectively. (**G**) Extended gel of Supplementary Fig. 6c (left) and Supplementary Fig. 8d right panel (right). The “G+A” lanes each indicate a G+A sequencing ladder generated using the corresponding nucleosome DNA template.

Supplemental Table 1 Single-molecule FRET events, SWRIC eviction of H2A from the nucleosome , eviction of H2A from the nucleosome independent of SWR1C, and SWRIC eviction of H3 from the nucleosome

[ATP] 100 uM									
Type of Events	Tethered Nucleosomes (#)	Priming Events (#)	Priming tau* (s)	95 % C.I. (s)	Median eviction (s)	Stdev (s)	Release Events (#)	Median Release (s)	Stdev (s)
Distal First	939	164	51	44.1-60.0	2.5	2.1	NAN	NAN	NAN
Proximal second	939	69	90	72.3-116.2	2.5	1.8	69	65	32.0
Proximal First	939	40	36	27.1-50.5	2	1.0	NAN	NAN	NAN
Distal second	939	10	71	41.4-147.45	3.5	0.9	10	18	5.8
Single Events	939	352	51	46.6-57.4	2.5	1.5	352	63	21.2
Aggregate Events	939	635	55	51.0-59.6	2	0.6	431	96[#]	68.0-120.5[##]
**[ATP] 5 uM**									
Type of Events	Tethered Nucleosomes (#)	Priming Events (#)	Priming tau* (s)	95 % C.I. (s)	Median eviction (s)	Stdev (s)	Release Events (#)	Median Release (s)	Stdev (s)
Distal First	940	156	73	62.7-85.9	3	1.7	NAN	NAN	NAN
Proximal second	940	73	112	90.4-143.2	2.5	2.1	73	88	51.5
Proximal First	940	15	39	24.7-69.3	2	2.2	NAN	NAN	NAN
Distal second	940	10	99	54.7-228.4	3	3.5	8	25	16.7
Single Events	940	315	64	58.2-76.6	3	2.2	315	92	61.0
Aggregate Events	940	569	73	67.2-79.3	3	0.6	398	136[#]	114-166[##]
**[ATP] 0.5 uM**									
Type of Events	Tethered Nucleosomes (#)	Priming Events (#)	Priming tau* (s)	95 % C.I. (s)	Median eviction (s)	Stdev (s)	Release Events (#)	Median Release (s)	Stdev (s)
Distal First	865	85	70	57.5-88.1	3.5	2.0	NAN	NAN	NAN
Proximal second	865	34	137	100.4-197.2	2.5	1.1	34	112	67.7
Proximal First	865	13	70	43.4-131.5	2.5	2.4	NAN	NAN	NAN
Distal second	865	4	139	63.5-511.1	3	1.3	4	88	47.2
Single Events	865	189	94	82.1-109.2	2.5	4.4	189	134	125.0
Aggregate Events	865	325	92	82.8-102.9	2	0.7	227	219[#]	204.5-240[##]
**[ATP] 0 uM**									
Type of Events	Tethered Nucleosomes (#)	Priming Events (#)	Priming tau* (s)	95 % C.I. (s)	Median eviction (s)	Stdev (s)	Release Events (#)	Median Release (s)	Stdev (s)
Distal First	962	66	132	105.2-170.7	4	2.6	NAN	NAN	NAN
Proximal second	962	2	151	54.2-1246.9	3.5	0.0	2	74	73.5
Proximal First	962	22	54	37.1-86.5	3	3.8	NAN	NAN	NAN
Distal second	962	0	NaN	NaN	NaN	NaN	0	NaN	NaN
Single Events	962	15	90	57.9-162.3	2.5	2.0	15	138	150.0
Aggregate Events	962	147	110	94.9-131.3	3	2.7	17	179[#]	149.5-331[##]

*tau = lifetime exponential fit of distribution, C.I. = 95 % confidence interval of exponential fit, [#]Half-life estimate Kaplien-Mier survival curve, [##]Half-life 95 % confidence interval

Supplemental Table 2 Single-molecule FRET events, eviction of H2A from nucleosomes independent of SWR1C, and SWRIC eviction of H2A from H3 and H2A fluorescently labeled nucleosomes

Type of Events	Tethered Nucleosomes (#)	Priming Events (#)	Priming tau* (s)	95 % C.I. (s)	Median eviction (s)	Stdev (s)	Release Events (#)	Median Release (s)	Stdev (s)
**Nucleosome (-)SWR1C [ATP] 100 uM**									
Distal First	567	45	89	66.5-130.7	3	2.9	NAN	NAN	NAN
Proximal second	567	4	126	38.3-345.7	3	1	4	78	64.2
Proximal First	567	14	58	41.3-87.2	3	1.2	NAN	NAN	NAN
Distal second	567	0	NaN	NaN	NaN	NaN	0	NaN	NaN
Single Events	567	21	85	41.8-118.4	2	1.7	21	64	92.5
Aggregate Events	567	84	88	52.0-122.2	3	2.3	25	119#	84.4-180.71##
**Double-label nucleosome [ATP] 0 uM**									
Type of Events	Tethered Nucleosomes (#)	Priming Events (#)	Priming tau* (s)	95 % C.I. (s)	Median eviction (s)	Stdev (s)	Release Events (#)	Median Release (s)	Stdev (s)
First Events	525	47	67	37.5-131.7	3	1.7	NAN	NAN	NAN
Second Events	525	2	144	51.8-1191.1	3	1.8	2	96	102.9
Single Events	525	18	57	35.4-103.7	2	1.1	18	76	67.3
Aggregate Events	525	67	65	46.6-138.7	3	2.8	20	125#	81.2-219.7##
**Double-label nucleosome [ATP] 100 uM**									
Type of Events	Tethered Nucleosomes (#)	Priming Events (#)	Priming tau* (s)	95 % C.I. (s)	Median eviction (s)	Stdev (s)	Release Events (#)	Median Release (s)	Stdev (s)
First Events	558	123	47	32.7-99.4	3	1.6	NAN	NAN	NAN
Second Events	558	36	83	33.1-228.5	3	0.4	36	38	52.5
Single Events	558	164	52	40.0-84.9	3	1.3	164	57	26.6
Aggregate Events	558	323	56	49.9-71.2	3	2.6	200	61.7#	53.9-71.3##

*tau = lifetime exponential fit of distribution, C.I. = 95 % confidence interval of exponential fit, #Half-life estimate Kaplien-Mier survival curve, ##Half-life 95 % confidence interval

Supplemental Table 3. Unwrapping events during priming phase				
	# Nucleosomes	#events	Freq.(sec^-1)	mean dwell (sec)
(-)ATP	491	152	0.0016	1.49 +/- 2.32
(+)ATP	487	234	0.0038	3.4 +/- 5.8
Nucleosome (-) SWR1C	73	5	0.0005	1.7 +/- 0.58
DoubleLabel (-)ATP	65	8	0.0011	1.2 +/- 0.44
DoubleLabel (+)ATP	78	21	0.0024	2.2 +/- 1.9

Supplemental Table 4. Single-molecule colocalization of H2AZ-Cy3b with SWR1C-nucleosome

# tethered nucleosomes	#events	time for 50% bound (s)	alpha* (C.I. 95%)	Dwell tau1* (C.I. 95%) (s)	Dwell tau2* (C.I. 95%) (s)	<Dwell>** (s)	# Depo. events	median arrival before Depo. (s)	Delay between binding and deposition (s)
307	1136	10	0.78 (0.73-0.82)	11 (9.7-11.9)	117 (91.0-155.9)	34	29	75+/-40	38+/-20

*Values correspond to output from fitting dwelltime distribution to a double exponential, alpha represents fraction of tau1, 95% Confidence interval obtained from bootstrap analysis. **corresponds to average dwelltime = alpha*tau1+(1-alpha)*tau2

**Supplemental Table 5 Oligonucleotides**	
oAM200	5' /5Biosg/CCAGTTACCTTCGGAAAAAGAGTTCAGTGCTTGGTAGTCGATCTCAACAGCGGTAAGATCC
oTG415	5' GGATCT/iCy3N/ACCGCTGTTGAGATC
oTG416	5' AACTCT/iCy5N/TTTCCGAAGGTAACTGG
biotTEG_117_w_N	5’ 5BiotinTEG/GAATTGTAATACGACTCACTATAGGGCG
ATTO647_4_s_N	5’ 5ATTO647N/CGCCCTGGAGAATCCCGGT
77_w_N	5’ GTACCCGGGGATCCTCTAGAGTG
0_s_N	5’ CTGGAGAATCCCGGTGCCGA
